# Supplementary material for: SMYD3 Promotes Cell Cycle Progression by Inducing Cyclin D3 Transcription and Stabilizing the Cyclin D1 Protein in Medulloblastoma
Source: Cancers (Basel). 2022 Mar 25;14(7):1673. doi: 10.3390/cancers14071673 (PMC8997160; doi:10.3390/cancers14071673)
Supplement: Supplementary file 1 [file cancers-14-01673-s001.zip › cancers-1595035-supplementary.pdf]

Supplementary Materials and Methods

Immunocytochemistry and Immunohistochemistry

For immunocytochemistry (ICC), cells grown in three-well chamber slides (Ibidi, Fitchburg, WI) were washed with PBS, fixed, permeabilized with ice-cold methanol, and blocked with 2% BSA in PBS. Cells were incubated with primary antibodies overnight at 4°C, washed with PBS, and incubated with fluorescent-labeled, species-specific secondary antibodies (Alexa Fluor®) for 1 h at RT. Before mounting, the slides were washed with PBS and incubated for 30 min in the dark with 1:100 dilution of 4'-6-Diamidino-2-phenylindole (DAPI) for nuclear staining. Slides were examined using Olympus BX61 confocal microscope with a 60-Å oil immersion objective. SPOT basic and SPOT advanced software was used to deconvolute Olympus BX61 images. Immunohistochemistry (IHC) was performed using an MB tissue array (Cat# BC17012b). Sections were deparaffinized in xylene and rehydrated in graded ethanol solutions. Antigen retrieval was carried out with 10 mM citrate buffer (pH 6) at boiling temperature for 60 min and permeabilized in 0.1% Triton-X100. The tissue sections were incubated with primary antibodies for SMYD3 overnight followed by 1 h incubation with horseradish peroxidase-conjugated secondary antibodies, 3,3′-diaminobenzidine (DAB) peroxidase substrate (Sigma) solution, and counterstained with hematoxylin for mounting. The images were captured with an Olympus BX61 fluorescent microscope attached with a CCD camera. Densitometry quantitation was determined using Image J software (NIH, Bethesda, MD).

Mass spectrometry

For the LC/MS, the enzymatically digested samples were injected onto a capillary trap (LC Packings PepMap) and de-salted for 5 min with a flow rate of 3 µl/min of 0.1% v/v acetic acid. The samples were loaded onto an LC Packing® C18 Pep Map nanoflow HPLC column. The elution gradient of the HPLC column started at 3% solvent A, 97% solvent B, and finished at 60% solvent A, 40% solvent B for 30 min for protein identification. Solvent A consisted of 0.1% v/v acetic acid, 3% v/v ACN, and 96.9% v/v H_2O. Solvent B consisted of 0.1% v/v acetic acid, 96.9% v/v ACN, and 3% v/v H_2O. LC-MS/MS analysis was carried out on a hybrid quadrupole-TOF mass spectrometer (QSTAR XL, Applied Biosystems, Framingham, MA). The focusing potential and ion spray voltage was set to 225 V and 2400 V, respectively. The information-dependent acquisition (IDA) mode of operation was employed in which a survey scan from m/z 400–1800 was acquired followed by collision-induced dissociation (CID) of the four most intense ions. Survey and MS/MS spectra for each IDA cycle were accumulated for 1 and 3 seconds, respectively. The obtained spectra were analyzed using scaffold (™) software version 3_00_07 (Portland, OR). The cut-off for these putative identified proteins were set to 99 % identity. Gel bands were excised with a razor blade and placed in microcentrifuge tubes. The gel piece was destained with 100 µl of gel destaining solution followed by washing with 400 µl of water. Then the gel pieces were dehydrated with 400 µl of 100% acetonitrile for 10 min and dried by vacuum. 100 µl of 100 mM DTT was added to the gel pieces and then incubated for 45 min at 55^0C. Next, 100 µl of 55 mM iodoacetamide was added and incubated for 30 min at RT in the dark. Gel pieces were then washed and dehydrated in a vacuum. Gel enzyme

solution was added and incubated for 1 h. Excess enzyme solution was removed and 25 mM ammonium bicarbonate was added prior to incubation at 37^{0}C overnight. The supernatant containing peptides were then transferred to a new microcentrifuge tube. The samples were desalted prior to LC-MS. Protein samples were then added to the affinity matrix (washed with PBS) and unbound or non-specifically bound proteins were eluted from the matrix. The bound proteins were eluted using 0.1 M glycine (pH 2.5). The eluted phosphopeptides were enriched by the addition of TIO_2 or IMAC using a combi syringe. The peaks were observed in Fourier (FT)-ion-cyclotron resonance (ICR) mass spectrometer (11 Tesla LTQ-FT ultra (Thermo Fischer Scientific, Waltham, MA).

Real-time PCR and RNA-Seq

Total RNA was isolated using Qiazol reagent (Qiagen, Germantown, MD). 2 µg of RNA was used to prepare complementary DNA using a cDNA synthesis kit (Bio-Rad). Reverse transcription reaction was carried out in a thermal cycler for 30 min at 55 ^{0}C followed by 5 min at 85 ^{0}C. The mRNA levels were examined by real-time PCR using CFX96 Real Time System (Bio-Rad). The analysis was performed in triplicates using SYBR green PCR master mix (Bio-Rad). The PCR conditions were 95 ^{0}C for 10 minutes, followed by 40 cycles at 95 ^{0}C for 10 seconds and 60 ^{0}C for 30 seconds. The expression level of target genes was normalized with actin values. RNA sequencing was performed using an Illumina GAllx sequencer with a Solexa sequencing primer. Two FASTQ files, one with all reads from shSMYD3 treated cells and the second one with all reads from control cells, were output from the high throughput sequencing. The files were converted to SCARF format, and any poor-quality bases were trimmed from the 3′-ends of the reads prior to alignment. BOWTIE was then used to align both the experimental and control files to the current version of the human genome NCBI V36. Aligned reads were then annotated with the gene name and whether the read occurred in the 3′-UTR or not. Counts for reads that occurred in annotated genes within the 3′-UTR and contained the seed sequence for treated samples were compared with control data. Fold change was then calculated as the number of reads in shSMYD3 treating condition/number of reads in the control condition. For calculation purposes, the number of reads in the control condition was set to 0.5 for any genes that were absent in the control.

Chromatin immunoprecipitation (ChIP)

ChIP seq analysis was performed by Array star (Rockville, MD). Briefly, 10 ng of DNA samples were prepared for Illumina sequencing in the following steps: 1) DNA samples were blunt-ended with T4 DNA polymerase and Klenow polymerase; 2) a dA base was added to the 3' end of each strand by Klenow (exo minus) polymerase; 3) Illumina's genomic adapters were ligated to the DNA fragments; 4) PCR amplification was performed to enrich ligated fragments; 5) Enriched product of ~200-1500bp was cut out from the gel and purified with QIAquick Gel Extraction Kit. The completed libraries were quantified by Agilent 2100 Bioanalyzer. The libraries were denatured with 0.1 M NaOH to generate single-stranded DNA molecules, captured on Illumina flow cell, amplified *in situ*. The libraries were then sequenced on the Illumina HiSeq 4000 following the TruSeq Rapid SBS Kit protocol. After the sequencing platform generated the sequencing images, the stages of

image analysis and base calling were performed using Off-Line Basecaller software (OLB V1.8). After passing the Solexa CHASTITY quality filter, the clean reads were aligned to the human genome (UCSC HG19 using BOWTIE software (V2.1.0). Aligned reads were used for peak calling of the ChIP regions using MACS V1.4.0. Statistically significant ChIP-enriched regions (peaks) were identified by IP, using a p-value threshold of 10^{-5}. The peaks in samples were annotated by the nearest gene using the newest UCSC RefSeq database.

Clonogenic assay

For Clonogenic or colony formation assay, shSMYD3 treated and control cells were grown for 48 hrs. Viable cells were then counted and 1000 cells were seeded in 60 mm plates. The cells were then incubated for 10 days at 37°C in a 5% CO_2 incubator. All colonies were fixed with 4% paraformaldehyde and stained with crystal violet. Colonies were quantified by manual inspection

MTT, Cytotoxicity, Viability and Caspase 3/7 assay

Cell viability was determined by MTT assays (Sigma, St. Louis, MO). D458, D425, and NHA cell lines were seeded in 96-well plates at a concentration of 5×10^3 cells/well. D458 cells were transfected with/without SMYD3-KO plasmids in the presence or absence of recombinant cyclin D1+CDK4 or cyclin D3+CDK4 complexes for 3 and 5 days. The D425, D458, and NHA cell lines were treated with increasing concentrations of BCI-121 (0, 10, 20, 40, 80, 100 µM) for 5 days. After treatment, the medium was replaced with 100 µL sterile MTT solution per well and cells were incubated for 2 h in a cell culture incubator at 37°C. The MTT crystals were solubilized in DMSO (Sigma, St Louis, MO, USA) for 30 minutes. Absorbance was read using a SpectraMax M5 spectrophotometer (Molecular Devices, Sunnyvale, CA) at 550 nm. The cell viability/cytotoxicity and caspase 3/7 assay was investigated with the HTS ApoTox-Glo™ Triplex Assay Kit (Promega), according to the manufacture's protocol. Briefly, the cells were treated with the desired shRNA/compound and then 20 µl of viability/cytotoxicity reagent was added to all the wells and briefly mix for 30 sec by shaking. Cells were incubated for 1 h at 37°C. Fluorescence was measured at 400Ex/505Em for viability and 485Ex/520Em for cytotoxicity. Caspase-Glo 3/7 reagent was then added to all wells and incubated for 1 h at RT. Changes in apoptosis were determined by comparing the luminescence of treated samples to controls.

Lactate dehydrogenase (LDH) detection

LDH released from apoptotic cells or dead cells was measured using CytoScan LDH cytotoxicity Assay (G-Biosciences, MO, USA) according to the manufacturer's instructions. 50 µL of conditioned media from the control and BCI-121 treated cells were mixed with 50 µL of reconstituted substrate mixture in a 96-well plate. The plate was incubated away from light at 37°C for 2 hours. 50 µL of stop solution was added to terminate the reaction. The assay was quantified by measuring absorbance at 490 nm using a SpectraMax M5 spectrophotometer (Molecular Devices, Sunnyvale, CA).

Table S1: Mass spectrometry analysis of immunoprecipitated proteins using anti-SMYD3 antibody

Gene	Pathway/function	Peptide sequence/Methylation
CDC5L	DNA Repair, Dynein, Dynactin, RAD1	1.DKLNINPEDGMADYSDPSYVKQMERESR ; K2me2 K21me3<br><br>2.ELEEREIDDTYIEDAADVDARK; K22me3
EP300	Histone acetyl transferase	1.SSLSRGNKKKPGMPNVSNDLSQKLYAT MEKHK; K10me3 K32me2<br><br>2.NHDHKMEKLGLGLDDESNNQQAAATQS PGDSRR; K5me1 K8me1<br><br>3.AENVVEPGPPSAKRPKLSSPALSASASDG TDFGSLFDLEHDLPDELINSTELGLTNG GDINQLQTSLGMVQDAASK; K16me3 K76me1<br><br>4.HHVETRWHCTVCEDYDLCITCYNTKNH DHKMEK; K33me1
RALY	RNA-binding protein Raly	1.R.AAVLGENGRVLAGQTLDINMAGEPKP DRPK.G<br><br>2.ANPDGKKK; K6me3 K8me1<br><br>3.YKNLMARALYDNVPECAEELAFRK; K2me3 K24me2
CDK8	Wnt/b-catenin, repressed E2F1, decreased E-cadherin and MMP-7	1.RKDGKDDK; K2me3 K8me3
CCNB2	Interact with CDK1	1.AGEVDVEQHTLAKYLMELTLIDYDMVH YHPSKVAAAASCLSQKVLGQGKWNL K; K13me3 K53me1<br><br>2. NVVKVNENLTKFIAIKNK; K4me3 K!6me2 K18me2
CCNB3	Promoting anaphase-promoting complex/cyclosome (APC/C) function	1. ESLDLQEKPSIKKETLLKKPLALK; K8me3 K12me3 K13me3 K!8me1 K19me3 K24me2
CCND1	Interacting with various CDK, G1/S phase	1.EVLPSMRKIVATWMLEVCEEQKCEEEVF PLAMNYLDRFLSLEPVKK; K22me3 K46me3

		2. CVQKEVLPSMRKIVATWMLEVCEEQK; K4me1 K12me3 K26me2
E2F3	Rb-E2F pathway	1.KSKNNVQWMGCSLSEDGGMLAQCQGLSKEVTELSQEEK; K1me2 K3me3 K28me1 K38me2<br><br>2. SKNNVQWMGCSLSEDGGMLAQCQGLSK; K2me3 K27me1
GA45G	Growth arrest and DNA damage-inducible protein GADD45 gamma	1. MTLEEVRGQDTVPESTARMQGAGK; K24me3
KDM1A	DDR DNA repair pathway, epigenetic	1. QVNMELAKIKQKCPLYEANGQAVPKEKDEMVEQEFNR; K8me1 K10me3 K12me2 K25me2 K27me3<br><br>2. IVKTQEELKELLNKMVNLK; K3me1 K9me2 K14me2
KDM2A	Downregulating Cyclin-dependent kinase inhibitor p21Cip1, upregulating PcG, EZH2, H2A ubiquitination	1. NSDGLGIKMPDPDFTVNDVKMCVGSR; K20me3 K8me1<br><br>2. WAVGIKDPQIR; K6me1<br><br>3. RKMEESDEEAVQAK; K2me1 K14me3
KDM2B	Upregulating CDK1, Cyclin A2 Cyclin B2	1. QTEATNAIAEMKYPKVKKYCLMSVK; K12me2 K25me3<br><br>2. KPSIDGFSSDSWLEMEEEACDQQPQEEEEK; K1me3 K30me1<br><br>3. YASNLPGSLLKEQKMNRDNK; K11me3 K14me1 K20me2<br><br>4. QVTKEGCEQFIAEMSVSVQFGQVEEK; K4me2 K26me1<br><br>5. TECGECHFCKDMKKFGGPGR; K!0me1 K!3me2 K14me3
KDM3B	Upregulating CDC123, cyclin D1	1. GEVDSNGSDGGEASRGPWKGGNASGEPGLDQRAK; K19me3 K34me3<br><br>2. SVLNKESHSPFGLDSFNSTAKVSPLTPK; K21me3 K28me1

		3. KSASDSGCDPASKKLK; K1me3 K14me3 K16me3<br><br>4. QGQPVLVSGVHKKLK; K12me3 K13me3 K15me3
KDM5A	Antagonizing pRB, suppresses CDKi P16, p21, p27	1. KLKLGADKSKELNKLAK; K1me3 K3me2 K17me1<br><br>2. KMDKPRKKKLK; K1me1 K8me3 K9me3 K11me2<br><br>3. SKELNKLAKKLAKEEER; K2me2 K6me3 K9me1 K10me1 K13me3<br><br>4. KILDLYALSKIVASK; K1me1 K10me3 K15me2
KDM5B	Upregulating E2F1 and E2F2	1. KKIKLSHPKDMNNFK; K2me3 K4me2 K9me3 K15me3<br><br>2. KLGVIDSERMDFELLPDDERQCVKCKTTCFMSAISCSCKPGLLVCLHHVK; K24me3 K26me2 K39me3<br><br>3. MGCPTPKCENEKEMKSSIK; K7me3 K12me2 K15me2 K19me1<br><br>4. QCVKCKTTCFMSAISCSCKPGLLVCLHHVKELCSCPPYK; K4me3 K6me3 K19me3 K39me1
H1.3	Histone 1	1. ASGPPVSELITK.A<br><br>2. SGVSLAALKK.A<br><br>3. ALAAAGYDVEKNNSR.I<br><br>4. SLVSKGTLVQTK.G
H1.5	Histone 1	1. ATGPPVSELITK.A<br><br>2. NGLSLAALKK.A<br><br>3. SLVSKGTLVQTK.G<br><br>4. AAKPKAAKAKKAAAKK.K ; K11me1; K8me2; K5me3 K10me3 K15me3 K16me3
H3.2	Histone 3	1. KSTGGKAPR.K; K6 me3
H4	Histone 4	1.GGKGLGKGGAKR.H; K3me1; K11me3
BRD4		1.EEVEENKKSKAKEPPPKK; K7me3 K8me3 K18me3

		2. KEPAPMKSKPPPTYESEEEDKCKPMSYEEK; K21me1 K23me3 K30me2<br><br>3. QKQEPKTPVAPKKDLKIK; K6me2 K13me1 K16me3 K18me3<br><br>4.MKGFSSSESESSSESSSSDSEDSETEMAPKSKK; K2me1 K30me3 K32me3 K33me3
M3K2	Regulates the JNK and ERK5 pathways by phosphorylating and activating MAP2K5 and MAP2K7	1.DSTGNVKLGDFGASKRLQTICLSGTGMKSVTGTPYWMSPEVISGEGYGRK; K7me1 K28me3
TAF1	Transcription initiation factor TFIID subunit 1; p53	1.LMPPPPPPPGPMKKDKDQDSITGEK; K13me2 K14me1 K16me3 K25me3<br><br>2.DKDQDSITGEKVDFSSSSDSESEMGPQEATQAESEDGK; K2me3 K38me2
OTX2	Homeobox protein;<br><br>CDKN, RB1, c-myc, E2F2	1.R.AQLDVLEALFAK.T<br><br>2.K.QPPYAVNGLSLTTSGMDLLHPSVGYPATPR.K
MENIN (MEN1)	Wnt signaling; regulates DNA repair	1. RESKPEEPPPPKKPALDKGLGTGQGAVSGPPR
HELZ	mTOR signaling; Class I RNA helicases, promotes proliferation	1. LVMTKVNAVYLLPVPKQKLVQTQGTKEK; K5me3 K16me3 K18me3 K26me1 K28me3
ERCC6	Encodes a DNA-binding protein that is important in transcription-coupled excision repair	1. GISGAPAGKKSR<br><br>2. ILNGEMQIFSGLIALRKICNHPDLFSGGPKNLKGLPDDELEEDQFGYWK<br><br>3. KITAKQKHLQAILGGAEVK, KKQGCNKR<br><br>4. QMENNFYKHKSKTKHHSVAEEETLEKHLRPK
MDM2	P53 signaling; regulates DNA repair, cell cycle, apoptosis	1.MCNTNMSVPTDGAVTTSQIPASEQETLVRPKPLLLKLLKSVGAQKDTYTMK; K45me3
DDX3X	ATP-dependent RNA helicase;<br><br>KLF4, WNT/b-catenin, DDX3/SNAIL/E-	1.EATKGFYDKDSSGWSSSK; K4me2, K9me3<br><br>2.GDRSGFGK; K8me2

	cadherin, KRAS/HIF1AMDM2/SLUG/E-cadherin, p53, WNT/b-catenin		

Table S2: Primers list

RT-PCR primers		
	**Forward primer**	**Reverse primer**
SMYD3	TTCACCATCTGTAATGCGGAGA	ACACAATCGAACAGTTGGGGT
Cyclin D1	CAATGACCCCGCACGATTTC	CATGGAGGGCGGATTGGAA
Cyclin D3	TACCCGCCATCCATGATCG	AGGCAGTCCACTTCAGTGC
Cyclin E1	GCCAGCCTTGGGACAATAATG	CTTGCACGTTGAGTTTGGGT
CDK1	CACCAGCGTTATATTTGCACAG	ATCTGCCAGCTTTAACTCCCC
CDK4	ATGGCTACCTCTCGATATGAGC	CATTGGGGACTCTCACACTCT
CDK7	GGAGCCCCAATAGAGCTTATACA	TCCACACCTACACCATACATCC
CDC25A	CTCCTCCGAGTCAACAGATTCA	CAACAGCTTCTGAGGTAGGGA
AKT1	CGACGTGGCTATTGTGAAGG	GATGATGAAGGTGTTGGGCC
mTOR	ATGCTTGGAACCGGACCTG	TCTTGACTCATCTCTCGGAGTT
Actin	GGACTTCGAGCAAGAGATGG	AGCACTGTGTTGGCGTACAG
**ChIP primers**		
Cyclin D1	GGAGGAAGGTGAGAAAGAGAAC	TGATAGAGGGTCCCTGAGTG
**Primers used for cloning**		
Cyclin D3 (RI)	AACTACTCGAGGCCGGTGAAGTAGGGTTTGA	AGTCAAAGCTTCCTCCAGCATCCAGTAAGCC
Cyclin D3 (RII)	AGTCAAAGCTTGACGATGTAGCAACCGTGGA	AACTACTCGAGGAAAACCGGACTACAGGGGC
Cyclin D3(RIII)	AGTCAAAGCTTGAAAGCCTCACGATGTAATGACT	AACTACTCGAGCCTGGGTGACTAGAGTCCGA

Supplementary Figures

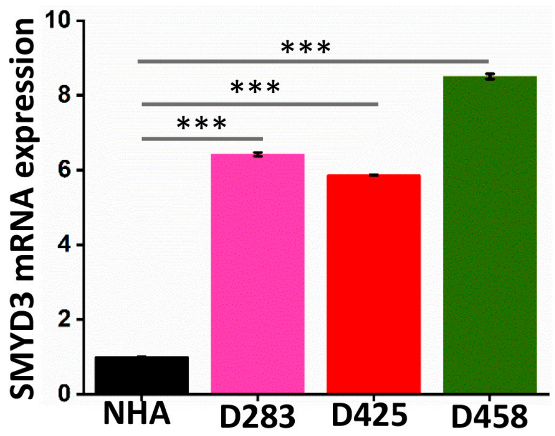

Figure S1: qRT-PCR analysis of SMYD3 transcript levels in the Group 3 MB cell lines D283, D425, and D458 compared to normal human astrocytes (NHA).

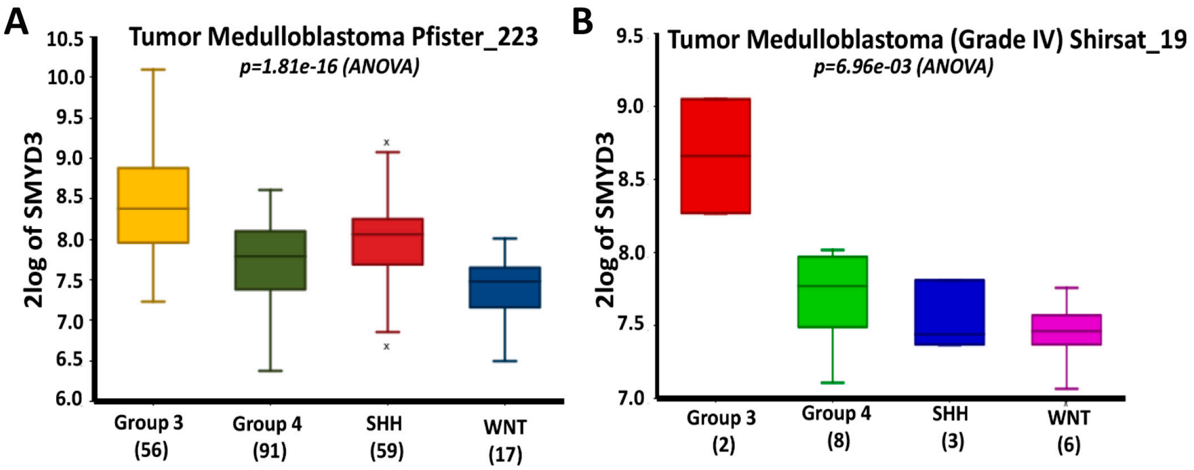

Figure S2: (A & B) Data mining using the Shirsat and Pfister datasets were used to determine SMYD3 expression among the four MB subgroups.

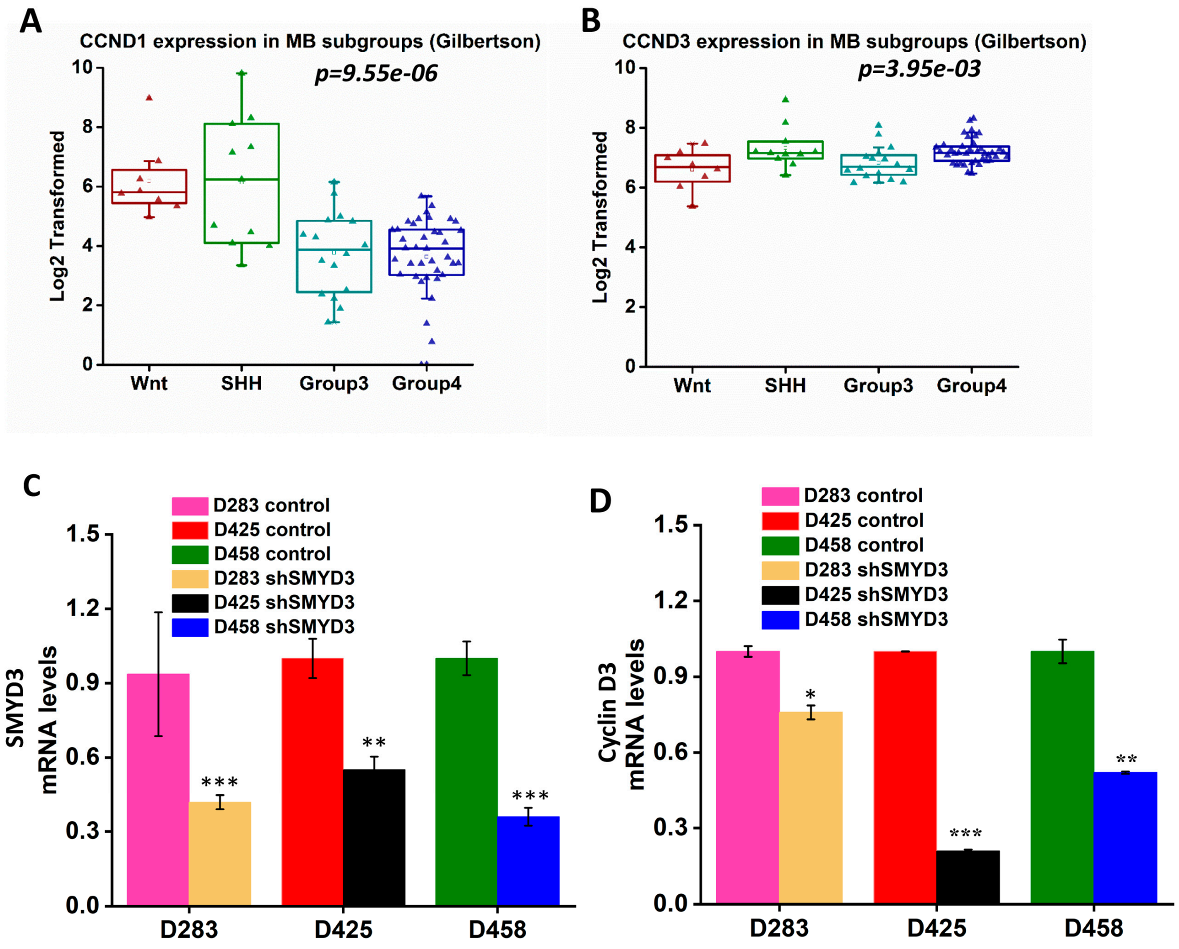

Figure S3: (A & B) Data mining studies using the Gilbertson MB patient dataset was used to determine the transcript levels of cyclin D1 and cyclin D3 in the four MB subgroups. **(C)** qRT-PCR analysis was used to confirm the downregulation of SMYD3 expression by shRNA for SMYD3 treated (shSMYD3) D283, D425, and D458 MB cell lines. **(D)** RT-PCR analysis to verify mRNA levels of cyclin D3 in control and shSMYD3 treated MB cells.

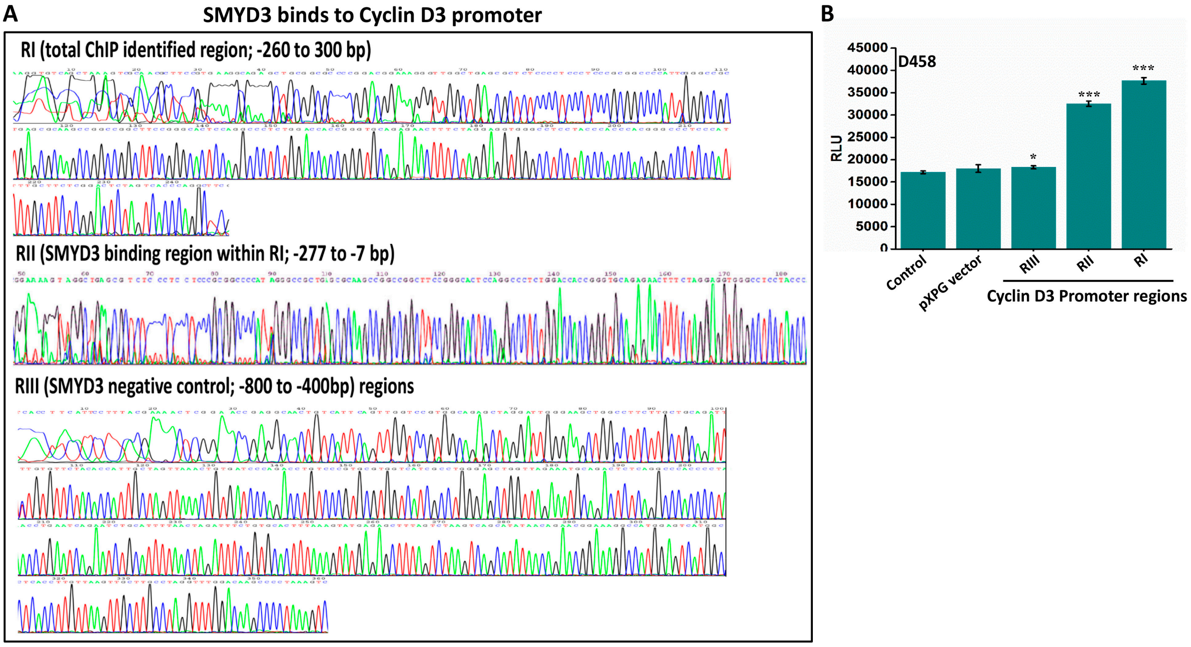

Figure S4: (A) Sequencing data of cloned reporter plasmids representing RI, RII, and RIII regions on the *cyclin D3* promoter. **(B)** Graph representing the luciferase activity of D458 cells transfected with regions RI, RII, and RIII.

A

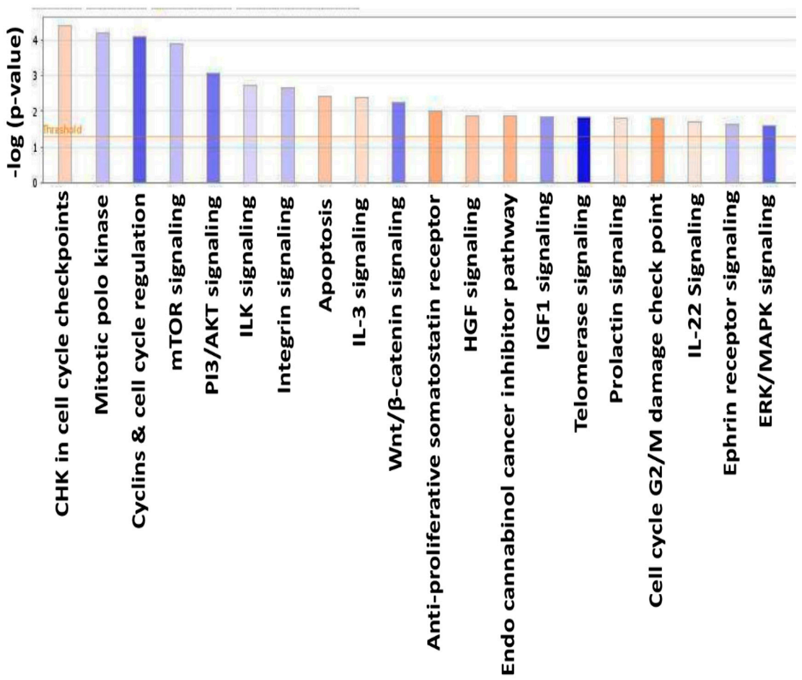

B

Activated genes	Fold change	Z-Score	p-value
CCND3	-2.07	1.0457	0.00486
CDK4	-1.08	0.577	0.0167
STAT4	-1.91	0.077	0.00443
KRAS	-1.05	-2.91	0.00793
PTEN	1.09	0.898	0.0266
MDM2	-2.94	1.445	0.0446
BRD7	1.07	-1.96	0.0699
MYC	-1.41	-1.867	0.143
MAP3K	-1.43	-2.157	0.176
RB1	1.23	-1.398	0.0014
ERK	-1.18	0.129	0.004
RAF	-1.56	-1.0803	0.00398
HDAC	-1.4	-0.0958	0.00415
BRAC1	1.35	-1.544	0.00466
TGFB	-1.18	-0.207	0.0101
MEK	-1.28	-2.639	0.0166
SMAD2	-1.18	-0.597	0.0297
CCND2	-1.01	0.687	0.034
ATM	1.24	-1.652	0.0342
E2F4	-1.2	-0.283	7.5E-07
E2F3	-1.07	1.221	0.0145
E2F2	1.03	-0.943	0.994

Figure S5: (A) The RNA-seq data of D283 cells were integrated into the Upstream Regulatory Analysis tool in the Ingenuity Pathway Analysis (IPA) software. IPA analysis showed significant inhibition of cyclins and cell cycle regulating molecules, PI3K/Akt, mTOR, and induction of apoptosis signaling. **(B)** Upstream Regulator Analysis tool within the IPA software was used to identify which genes were transcriptionally inhibited in SMYD3 knockdown cells.

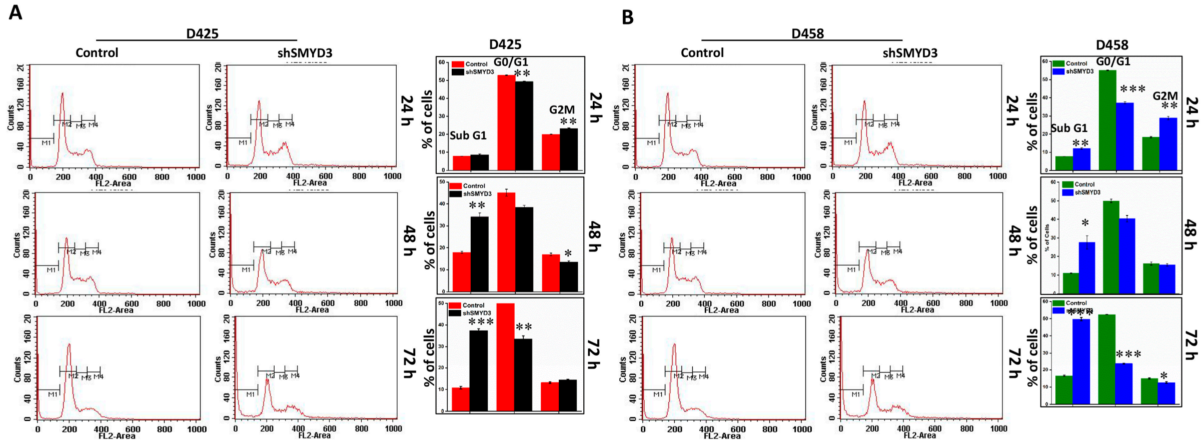

Figure S6: (A& B) Flow cytometry analysis of D425 and D458 cells 24, 48, and 72 h after shSMYD3 treatment. All analyses were compared to their respective control. Bar graphs representing data obtained from FACS analysis.

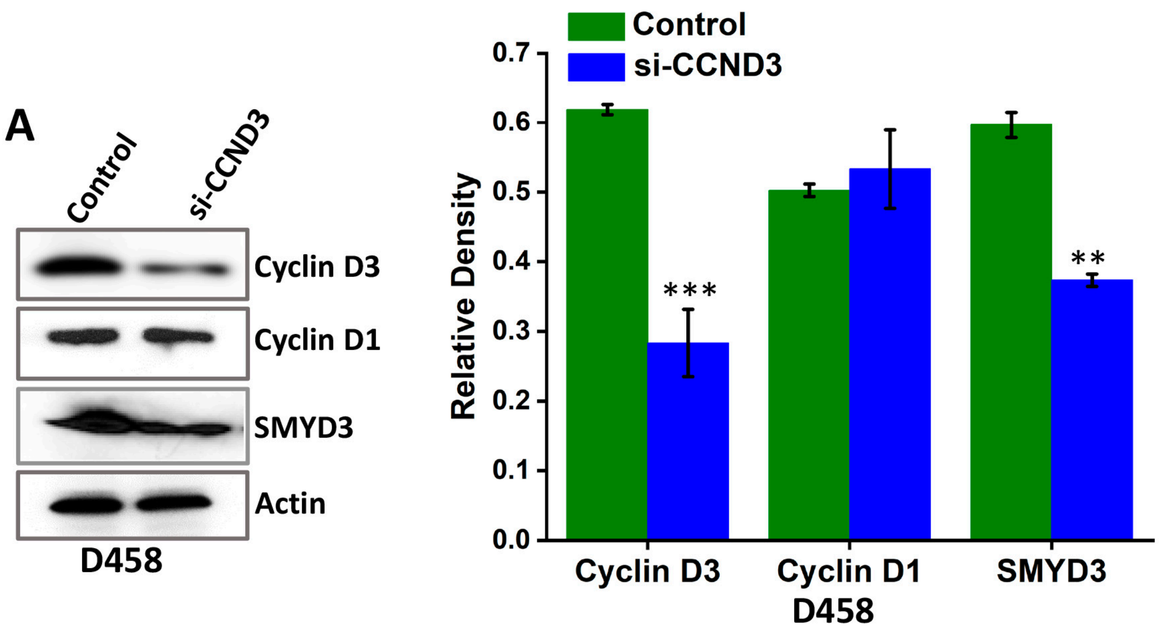

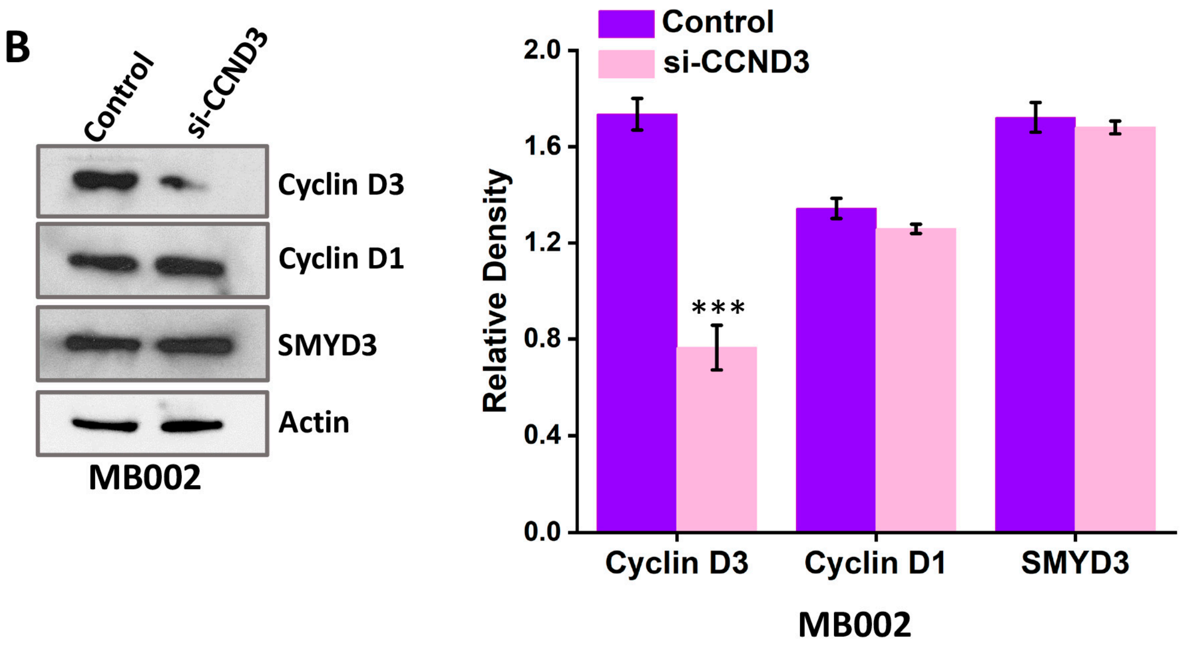

Figure S7: D458 and MB002 MB cells were transfected with siRNA targeting cyclin D3 (siCCND3). Immunoblot analysis of the control and siCCND3-treated MB cells. Actin was

used as the loading control. Graph depicts the densitometry analysis for the proteins in the western blot in MB cells.

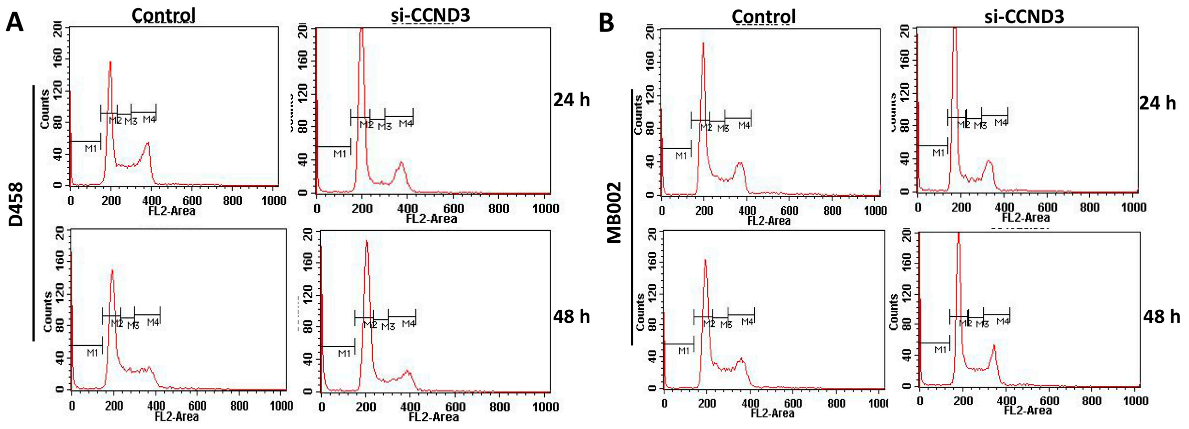

Figure S8: Flow cytometry analysis (raw data from FACS) of D458 and MB002 cells 24 and 48 h after siCCND3 treatment as compared with their respective control. The bar graphs are represented in the main manuscript Figure 9.

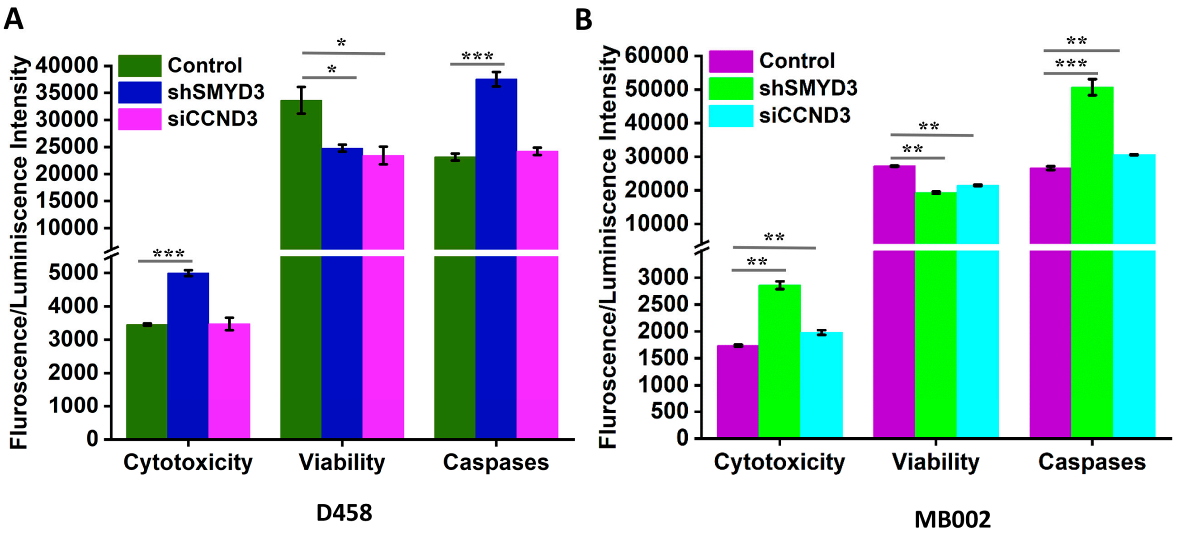

Figure S9: (A & B): Cytotoxicity, viability, and caspase 3/7 assay (ApoTox-Glo Triplex assay) showing the cytotoxicity/viability and apoptosis of D458 and MB002 cells treated with shSMYD3 and siCCND3 when compared to controls.

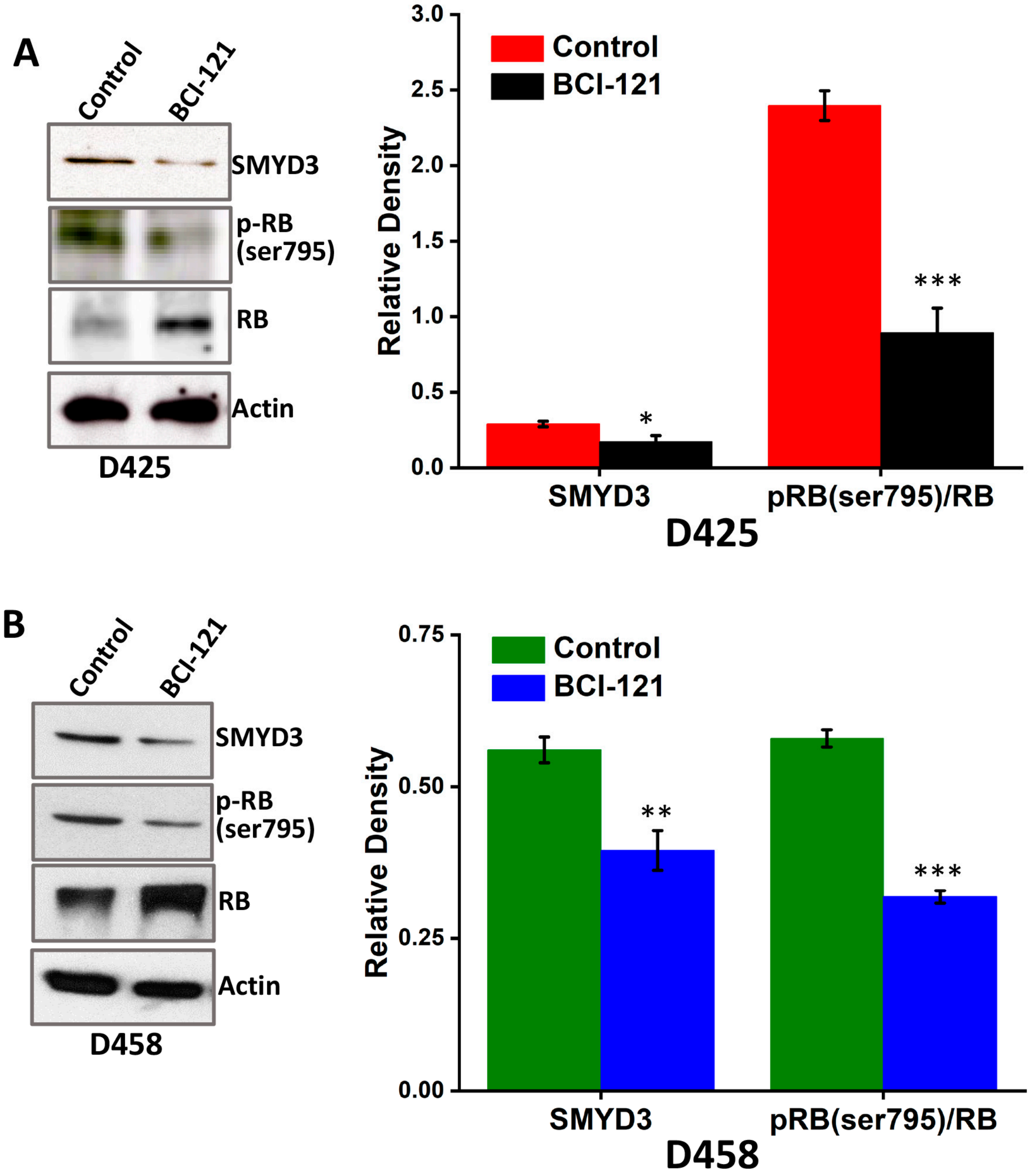

Figure S10: (A & B) Protein expression levels of SMYD3, pRB (Ser795), and RB in D425 and D458 cell lysates treated with or without BCI-121 (80 μM). Graph depicts the densitometry analysis for the proteins in the western blot in MB cells. The ratio of the relative level of phosphorylated (p) RB to total RB is plotted for each experimental condition.
